# Boldness and niche width structure spatial interactions in a natural rodent community

**DOI:** 10.1111/1365-2656.70320

**Published:** 2026-07-17

**Authors:** Jonas Stiegler, Annika Schirmer, Jana A. Eccard, Florian Jeltsch, Melanie Dammhahn

**Affiliations:** ^1^ Animal Ecology, Institute of Biochemistry and Biology University of Potsdam Potsdam Germany; ^2^ Plant Ecology and Nature Conservation, Institute of Biochemistry and Biology University of Potsdam Potsdam Germany; ^3^ Behavioural Biology, Institute for Neuro‐ and Behavioural Biology University of Münster Münster Germany; ^4^ Joint Institute for Individualisation in a Changing Environment (JICE) University of Münster and Bielefeld University Münster Germany

**Keywords:** individual niche differentiation, interspecific competition, intraspecific variation, niche theory, rodent community, space use, spatial interactions, species coexistence

## Abstract

1. Consistent among‐individual differences in behaviour can influence how individuals distribute in and use space, and thus influence competition within and between species, with potential consequences for species coexistence. These behavioural traits help characterize ecological niches, particularly under varying intensities of interspecific competition. While previous work examined behavioural type–dependent spatial interactions between pairs of ecologically similar species, here we extend those analyses to a full multi‐species community context, including newly analysed species.
2. In a natural rodent community of four co‐occurring species, we tested whether boldness predicts space use and spatial interactions within and across species of varying ecological similarity.
3. Boldness covaried positively with core area and home range size across all species, indicating consistent behavioural type–dependent differences in space use. In contrast, microhabitat use and neighbour density showed little association with boldness. Evidence for personality–dependent spatial structuring emerged primarily in metrics capturing spatial proximity and ecologically similar encounters, suggesting that behavioural effects on interactions are context dependent, being most evident among ecologically similar competitors and in distance‐based measures of spatial interactions.
4. Overall, our results suggest that boldness is most clearly linked to how individuals use space, whereas effects on spatial encounter structure within the community are weaker and appear to differ between the relatively more generalist and more specialist species in the community with respect to habitat use and diet. These findings support the idea that behavioural type–dependent space use can contribute to individual niche differentiation and may influence how potential interactions are distributed within ecological communities.

## Graphical abstract

We show that individual behavioural differences (boldness) structure spatial encounter patterns within a natural multi‐species community. By linking personality to space use and interaction structure, our study demonstrates how intraspecific variation can scale up to shape community‐level processes.

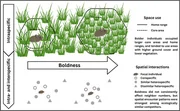

## 1 INTRODUCTION

Every species is embedded within a network of ecological interactions, yet understanding the mechanisms that enable species to organize into communities and coexist remains challenging. Classical theory suggests that interspecific differences in ecological niches (e.g. variation in resource use or habitat preference) facilitate coexistence by reducing direct competition (Chesson, 2000a; Hutchinson, 1959). On this basis, several mechanisms have been shown to reduce interspecific competition and promote stable coexistence through niche partitioning: species differences in their partitioning of resources (Hutchinson, 1957; Macarthur & Levins, 1967; Tilman, 1982), temporal or spatial partitioning of one resource (e.g. Chesson, 2000a, 2000b) or their density‐ or frequency‐dependent predation (e.g. Holt, 1984; Holt & Lawton, 1994; Holt & Polis, 1997). These mechanisms may act simultaneously within communities to stabilize species coexistence. Further, the concepts of interspecific competition and species coexistence mostly assume ecological niche homogeneity across individuals of a species or population (Colwell & Futuyma, 1971; Macarthur & Levins, 1967). This approach has been challenged conceptually with the argument that predictions of community ecology theory will improve by incorporating intraspecific trait variation (Hart et al., 2016; Violle et al., 2012; Yamauchi & Miki, 2009). This variation among individuals ultimately means that individuals within a species can differ in ecological niches within the larger species niche. Consequently, current ecological niche theory expands from species‐specific niche concepts to include individualized niches (Araújo et al., 2011; Bolnick et al., 2003, 2007; Costa‐Pereira et al., 2018; Trappes et al., 2022; Valen, 1965). However, empirical evidence linking individual behavioural variation to spatial interaction patterns within natural communities remains limited, particularly in systems where multiple ecologically similar species coexist.

Competition between community members of the same trophic level is assumed to be high if they are of similar size (Hutchinson, 1959) and fulfil similar roles within the community (i.e. are of the same functional type). More generally, competition is expected to increase with the degree to which species overlap in their use of limiting resources. Because species that are ecologically similar are also more likely to exploit similar resources, competition should increase with ecological similarity. Ecological similarity between species is inherently difficult to define (Abrams, 1983), but overlap between ecological niches, mainly of food resource use, is classically applied to assess the potential strength of competitive interactions between species. Such niche overlap calculations are often confined to single niche dimensions, for example diet. In our study, we assume species of similar body size, overlapping in crude dietary composition, space use requirements, and microhabitat use as ecologically similar.

Rather than focusing on niche specialization per se, recent work has emphasized how consistent behavioural differences among individuals influence ecological interactions and space use, thereby shaping encounter structure within communities (Bolnick et al., 2011; Schirmer et al., 2020; Toscano et al., 2016; Violle et al., 2012). This perspective links animal personality research directly to community ecology by identifying behavioural variation as a potential driver of interaction networks among co‐occurring species.

Although dietary overlap has historically dominated studies of intraspecific ecological variation (Araújo et al., 2011; Costa‐Pereira et al., 2019; Costa‐Pereira & Araújo, 2024; Harrison et al., 2017; Neves et al., 2021; Toscano et al., 2016), consistent behavioural differences among individuals can also structure how individuals use space and encounter competitors. Such repeatable behavioural variation, commonly termed animal personality (Gosling, 2001; Réale et al., 2007; Stamps & Groothuis, 2010), provides a mechanistic link between individual traits and ecological interactions. Recent work has increasingly demonstrated that individual behavioural variation is closely linked to intra‐ and interspecific interactions (Moran et al., 2017; Schirmer et al., 2020; Toscano et al., 2016). Personality traits can influence how individuals engage in competitive and spatial interactions by shaping movement behaviour, resource use, and spatial overlap with conspecifics and heterospecifics. At the same time, the social and competitive environment can feed back on the expression of personality traits, indicating a bidirectional relationship between behavioural types and interaction processes (Castellano et al., 2022; Wauters et al., 2019).

Building on these links between personality and ecological interactions, recent studies have combined both concepts, showing that consistent individual differences in behaviour facilitate individual niche specialization along various niche dimensions, that is dietary niches (Harris et al., 2020; Moran et al., 2017; Toscano et al., 2016), or spatial niches (Kerches‐Rogeri et al., 2020; Schirmer et al., 2019), resulting in non‐random, behavioural type–specific interspecific competitive interactions (Schirmer et al., 2020). Hitherto, most empirical studies focused on pairs of ecologically similar, directly interacting species (but see Meiden et al., 2023). In our study system, bank voles and striped field mice represent relatively more generalist species, exploiting a broader range of habitats and exhibiting a more flexible, omnivorous diet. In contrast, common voles and field voles are relatively more specialized on grassland habitats and grass‐dominated food resources (see Section 2). However, under natural conditions, each species is embedded in an ecological community and thus part of a nexus of interspecific interactions varying in type and strength. Hence, interactions within the whole community are to some extent influenced by individual behavioural variation within the community members (Meiden et al., 2023; Moran et al., 2017; Toscano et al., 2016). Thus, rather than the species‐specific traits alone determining species composition and dynamics, individual behavioural specialization most likely plays an important role in shaping natural communities (Costa‐Pereira & Araújo, 2024). Previous studies in a small mammal system demonstrated behavioural type–dependent spatial interactions within and between species pairs (Schirmer et al., 2019, 2020). These earlier findings suggest that behavioural types may differ in how they distribute spatial overlap among competitors. However, whether such personality‐dependent spatial structuring scales from species pairs to a broader community context remained unresolved. Here, we explicitly build on these findings but extend the analytical framework to a community context, including additional species and newly analysed data. Shyer individuals showed higher home range overlap with conspecifics compared to bolder individuals, while bolder individuals had more spatial overlap with heterospecific individuals compared to shy individuals (Schirmer et al., 2020). This difference in relative importance of intra‐ versus interspecific competition, covarying with behavioural traits (i.e. competitive syndrome, Erixon et al., 2024), between the behavioural types could facilitate species coexistence by decreasing species niche overlap and thus resource competition (Bolnick et al., 2003; Violle et al., 2012).

Here, we investigate how consistent individual behavioural differences structure spatial encounter patterns within and between co‐occurring small mammal species that vary in ecological similarity, thereby directly linking personality–dependent space use to interaction structure within ecological communities. Such behavioural mediation of ecological interactions, whereby consistent individual differences influence how individuals use space and encounter others, has been proposed as a key mechanism linking individual variation to community dynamics (Bolnick et al., 2011; Violle et al., 2012). More specifically, we examine whether personality–dependent differences in space use translate into systematic differences in spatial encounter structure within the community. We hypothesized that within‐species trait variation is most decisive in determining interactions between ecologically similar species because of higher competition. Thus, we address the possible importance of variation on the individual level for community interactions and species coexistence. By extending analyses from species pairs to a multi‐species community context, our study tests whether personality–dependent spatial structuring generalizes across a network of interacting species differing in ecological similarity.

As a study system, we used natural, free‐living communities of four co‐occurring, similar‐sized (20*−*30 g) microtine and muroid rodents in agricultural, central European landscapes. Within this community, the four species differ in ecological breadth. All species share a set of ground and avian predators and partially overlap in temporal activity patterns (Daan & Slopsema, 1978; Gliwicz, 1981; Jancewicz & Gliwicz, 2017; Kozakiewicz, 1987; Kozakiewicz & Boniecki, 1994). Therefore, ecological niches of all species should, to some extent, overlap since some resources are shared between all four species (i.e. predator‐free area, parts of the diet, and habitat), with modes of separation among the pairs including mobility, food type, and complexity of the habitat. To the extent that these shared resources are limiting, overlap in their use creates the potential for competitive interactions among individuals and species. Niche overlap is increasing within the species pairs of the same functional type (i.e. fewer modes of separation). We therefore expected ecological overlap to be greatest among conspecifics, intermediate among heterospecifics with similar habitat use and trophic ecology, and lowest among the more dissimilar species combinations. In this sense, ecological similarity in our study system can be interpreted along a gradient that also separates the relatively more generalist from the relatively more specialist species. Assessing competition and ecological similarity empirically is challenging (Abrams, 1983), resulting in the need to use proxies. In our study system, competition happens mainly indirectly via the exploitation of resources (Kozakiewicz & Boniecki, 1994; Myllymäki & Myllymaki, 1977). For this type of competition, space use reflects the extent to which individuals use overlapping portions of the habitat and resource landscape. Spatial overlap therefore provides a proxy for the opportunity for ecological interactions, including indirect interactions mediated through shared resource use, rather than a direct measure of competition strength. To address this, we combined capture‐mark‐recapture, behavioural assays, and automated radio telemetry to quantify individual boldness, space use and spatial overlap in a replicated field study.

Specifically, we asked: (1) how does individual behavioural variation covary with space use, including core area size, home range size, and microhabitat characteristics? Based on previous work (Dammhahn et al., 2022; Erixon et al., 2025; Schirmer et al., 2019, 2020), we expect that bolder individuals across the community occupy larger core areas and larger home ranges characterized by lower vegetation height and more ground cover compared to shy conspecifics. And, (2) how does boldness predict spatial overlap with conspecifics and heterospecifics, and does this vary with the ecological similarity of species? Here, considering the findings of Schirmer et al. (2020), we hypothesized that bolder individuals would occupy larger ranges and therefore differ systematically in spatial encounter probabilities across a gradient of competitor similarity, ranging from conspecifics (strongest competitors) to ecologically similar heterospecifics and ecologically dissimilar heterospecifics (weakest competitors; Figure 1). Under this framework, behavioural types are expected to differ not categorically in whom they overlap with, but in how they distribute encounter probabilities along this competition gradient. We hypothesize further that interactions with ecologically less similar heterospecifics are less strongly related to boldness because lower niche overlap should reduce the extent to which personality‐dependent spatial positioning modifies potential competitive encounters.

**FIGURE 1:**
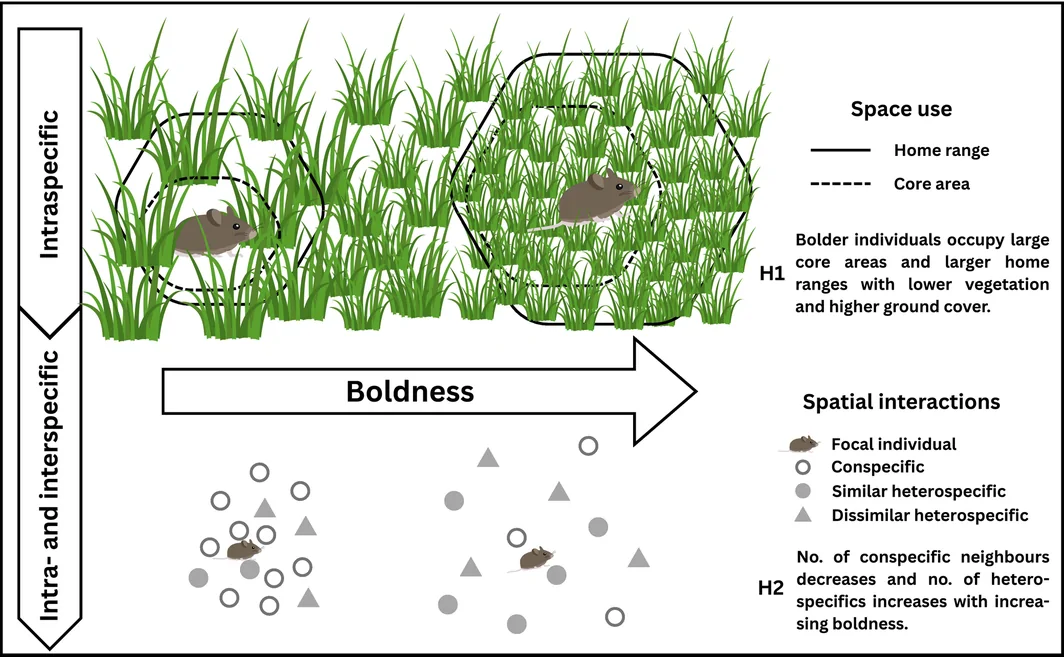
Schematic and graphical representation of the study predictions on (upper half) the expected interspecific differences in space use along a shy‐bold gradient. Exemplary space use is shown for a shy individual (upper left) and a bold individual (upper right), while the home range is depicted with a solid line, and the core area with a dashed line. Lower half: The expected intra‐ and interspecific number of individuals within a focal individual's vicinity, depending on boldness and ecological niche similarity. The symbols depict the distribution of home range centres of other individuals in the community, separated by species and ecological niche similarity. The scheme illustrates the prediction that behavioural types may differ in how encounter probabilities are distributed along this competition gradient.

## 2 MATERIALS AND METHODS

### 2.1 Study sites

The study was conducted at AgroScapeLabs in northwestern Brandenburg, Germany (53°21′56.2″ N, 13°48′17.3″ E; http://www.zalf.de/de/struktur/eip/Seiten/AgroScapeLab.aspx), an intensively farmed landscape with large fields interspersed with hedgerows and fallow lands. Fallow lands served as confined habitat islands for ground‐dwelling small mammals. We selected five sites (0.85–1.66 ha) for our study, which were separated from each other via barriers such as roads, agricultural fields, and human settlements, lowering the probability of exchange of study individuals between sites. In those areas, vegetation was heterogeneous, consisting of grasses, bushes, and scattered trees. Dominant taxa included Poaceae (40%), *Urtica* spp. (21%), and *Ballota* spp. (9%). Sites were visited consecutively from August to November 2016. All experimental procedures (capture‐mark‐recapture, behavioural testing, and VHF telemetry; see below) were conducted over continuous periods of 13 ± 3 days per site.

### 2.2 Capture‐mark‐recapture

We conducted intensive capture–mark–recapture (CMR) at each site using a grid of 55 Ugglan live traps (Special No. 2, Grahnab, Sweden), spaced 10 m apart. Traps were baited with rolled oats and apples, and pre‐baited in an inactive state for 24 h before activation. Activated traps were always checked after 8 h. Depending on weather conditions, traps were either checked twice daily (morning and afternoon) or only in the morning. Trapping was carried out over 9 ± 3 consecutive days per site. Individuals captured for the first time were temporarily marked with fur cuts. Upon first recapture, we performed individual difference tests with adults >17 g, excluding late‐gestation or lactating females, and permanently marked individuals with a passive integrated transponder (PIT, Euro I.D., trovan® ID100). We captured 75*–*103 rodents per site, corresponding to 151*–*260 rodents/ha densities. The two most abundant species were bank vole (*Myodes glareolus*, 40*–*157/ha) and striped field mouse (*Apodemus agrarius*, 22*–*74/ha), followed by common vole (*Microtus arvalis*, 14*–*84/ha) and field vole (*Microtus agrestis*, 13*–*23/ha). Occasional captures of yellow‐necked mice (*Apodemus flavicollis*, 6*–*16/ha) and wood mouse (*A. sylvaticus*, 0*–*10/ha) were excluded from this study due to small sample sizes. No individuals were ever trapped on grids other than the one from which they originated. Density estimates are based on minimum number alive per trapping grid area and should therefore be interpreted as conservative approximations rather than model‐based population estimates. More complex capture–recapture estimators were not applied because sampling duration per site was short and designed for behavioural rather than demographic inference, violating assumptions required for reliable survival‐based density estimation.

### 2.3 Study species and behavioural testing

We studied rodent communities comprising bank voles, striped field mice, common voles, and field voles. To assess how ecological similarity influences spatial behaviour, we grouped the four study species into two ecologically distinct species pairs, classifying species of similar body size, overlapping in crude dietary composition, space use requirements and microhabitat use as ecologically similar: The first pair, the microtine bank vole, and the muroid striped field mouse, share an omnivorous diet, large home ranges and occupy a wide variety of habitats including woodlands, shrubs, meadows, grassy areas, and hedges (Eccard et al., 2002; Holišová, 1971; Kozakiewicz & Boniecki, 1994). Both forage above ground and climb shrubs and trees. The second pair, common voles and field voles, both microtines, are mainly granivorous (Myllymäki & Myllymaki, 1977), inhabit grassy areas, have small home ranges, forage below ground, and use linear tunnel and runway systems for above‐ground movement (Schirmer et al., 2019; Szalay et al., 2000). Accordingly, we refer to bank vole and striped field mouse as the relatively more generalist species pair and the two *Microtus* species as the relatively more specialist pair. These pairings reflect relative positions along an ecological similarity gradient among species in this system rather than discrete taxonomic or functional categories. We consequently labelled individuals not belonging to the species pair of a given focus individual as dissimilar and summarized the ecological differences across four behavioural and ecological dimensions (Table S1).

For all species, interindividual differences in behaviour were quantified in situ using a mobile setup combining two established laboratory procedures: the dark–light and the open‐field test. Considering potential challenges of comparative animal personality research (e.g. Carter et al., 2013; White et al., 2020), we assumed that repeatably observed behaviours captured comparable latent personality dimensions across species, as they are similar in size, share the same predators, and perceive vegetation cover and height as a proxy of predation risk. Tests were conducted in comparable locations across study sites, and only on rainless days with low wind speed. Individuals were tested immediately after capture and re‐tested after 1*–*7 days. The test setup and procedure followed the routines described in Schirmer et al. (2019, 2020). In short, during the dark–light test, the individuals moved from the trap into an opaque plastic pipe (10.5 cm × 32 cm) with swing doors at both ends, connected to a round arena (1.30 m diameter, 30 cm height). One observer (AS) recorded the latency to stick the head out of the pipe (latency head) and to fully exit the pipe (latency body; excluding tail). If an individual did not exit within 300 s, it was gently pushed out, and the latency was set to 300 s (12% of all tests). Once the individual entered the arena, the open‐field test began. The arena (diameter 1.3 m) was visually divided into an exposed centre and a border area along the walls, each covering approximately equal surface area, representing high‐ and low‐risk areas, respectively. Over 300 s, we quantified (i) latency to enter the centre for the first time, (ii) the number of crossings into the centre, (iii) the number of jumps, (iv) the number of arena sections entered (arena virtually divided into 16 s to quantify exploration), and (v) activity, recorded every 10 s as a binary measure (active vs. inactive based on movement or scanning behaviour; see Schirmer et al., 2019).

For each behavioural variable, we estimated repeatability using the rptR package (Stoffel et al., 2017, Table S2). All repeatable variables (*R >* 0) were entered into a principal component analysis (PCA) performed on the pooled dataset across all four species. We included inverted latency measures (300 *−* latency to head emergence, body emergence, and first centre entry), as well as crossings, sections entered, and activity. PCA was conducted on the correlation matrix of these behavioural variables using oblimin rotation, allowing components to be correlated. Scree plots and parallel analysis indicated a two‐component solution, corresponding to exploration and boldness (Tables S2 and S3). To verify that behavioural axes were comparable across species, we additionally performed a grouped PCA using species identity as the grouping factor. Loadings were highly similar across species (>0.9 similarity), indicating that behavioural variables contributed consistently to the same latent components across taxa. This suggests that pooled PCA scores represent homologous behavioural axes rather than species‐specific constructs. Previous work in this study system showed that boldness, rather than exploration, consistently covaried with space use and spatial interaction metrics (Schirmer et al., 2019, 2020). In addition, boldness is conceptually more directly linked to risk‐taking and predator avoidance, and was therefore expected to be more closely associated with spatial positioning and space‐use decisions than exploration, which primarily reflects movement within a given space. We therefore focused our main analyses on boldness as the behavioural trait most directly linked to spatial behaviour. Exploration scores are reported in the Supporting Information (Tables S2 and S3) to document species‐specific personality structure and confirm that results are robust to trait choice. Individual boldness scores were used as repeated measures in subsequent analyses.

### 2.4 Movement tracking and spatial interaction analysis

#### 2.4.1 Microhabitat characteristics

To assess general space use patterns across species, we quantified ground cover 10 cm above ground and maximum vegetation height within spatial ranges. Percentage ground cover was estimated from photos taken of 1 m^2^ around each trap location within the trapping grid, while maximum vegetation height was measured directly in the field within the same area. We interpolated both variables with an inverse weighting of the distance between points using QGIS 2.18.14 (QGIS Development Team, 2021) to estimate ground cover and maximum vegetation height for the whole trapping grid.

#### 2.4.2 Automated radio telemetry

Tracking data for bank voles and striped field mice were previously analysed in Schirmer et al. (2019, 2020) for species‐specific questions. In the present study, we reanalysed these data within a unified community framework and combined them with newly analysed data for field and common voles. While some space‐use patterns for bank voles and striped field mice overlap with previously published results, all interspecific encounter analyses and all analyses for field and common voles are newly presented here in a community‐level framework.

We installed an automated radio tracking system surrounding the trapping grids at each study site to assess the space use of a subset of individuals on a fine spatial scale. Each consisted of eight omnidirectional antennas at ground level connected to automatic receiving units (ARU; JDMC Corp, Illinois, US; four antennas/ARU, more details in Schirmer et al., 2019, 2020). Individuals were fitted with VHF radio transmitters on collars (1.1 g; BD‐2C, Holohil Systems Ltd., Canada), emitting data for four consecutive days. Individuals were selected for tagging based on body mass (>20 g, i.e. transmitter weight ≤5% of body mass) and recapture probability (>2 captures prior collaring), excluding females in late gestation. Tracking started at each site directly after the last individual difference test was performed. In total, we tracked 21 bank voles (9 females, 12 males), 15 striped field mice (6 females, 9 males), 13 field voles (4 females, 9 males), and 5 common voles (1 female, 4 males). Inference for common voles is therefore based on limited telemetry data, and we interpret results for this species with caution. Detailed analyses of tracking data on bank voles and striped field mice are in Schirmer et al. (2019, 2020). For each individual, we sampled an average of 96 locations per day with a mean location error of 9.4 ± 7.3 m, influenced by vegetation density and humidity.

#### 2.4.3 Spatial interaction analyses

Spatial interaction analyses were restricted to individuals with empirically estimated telemetry‐based core areas and home ranges (*n* = 54). Activity centres of all individuals present during the same sampling period were derived from capture–mark–recapture (CMR) data and used to quantify neighbouring individuals within these empirically defined space‐use areas (*n* = 399). For all radio‐tracked individuals, we estimated core area size (as 50% kernel) and home range sizes (as 95% kernel) using the R package adehabitatHR (Calenge, 2006), and assessed microhabitat characteristics within these ranges based on interpolated ground cover and maximum vegetation height. For all recaptured individuals, we calculated a mean trapping point as a proxy for the centre of spatial activity (4.3 ± 3.9 captures/individual; mean ± SD). The suitability of mean trapping locations as estimates of activity centres was evaluated in a sensitivity analysis presented in the Appendix of Schirmer et al. (2020).

We quantified within‐ and between‐species spatial interaction opportunity using empirically estimated space‐use areas derived from radio‐tracking data. For each tracked individual, we calculated its core area (50% kernel) and home range (95% kernel) from telemetry locations (see above). To assess spatial proximity to other individuals, we used concurrent CMR data to estimate activity centres (mean trapping point across captures) for all individuals present at the same site and sampling period. For each tracked focal individual, we counted the number of other individuals whose activity centres fell within its core area and within its home range. To quantify covariation between behavioural traits and spatial interaction metrics at the community level, we fitted multivariate covariance models that included species as a fixed effect.

This approach allowed us to estimate personality–space‐use relationships on a shared trait scale across species while avoiding overparameterization that would arise from fully species‐specific interaction models given the limited telemetry sample sizes for some taxa. Individuals were classified as conspecifics or heterospecifics, and heterospecifics were further categorized as belonging to ecologically similar or dissimilar niche groups relative to the focal species. Analyses were restricted to tracked individuals because only for these individuals space use could be directly quantified.

#### 2.4.4 Statistical analyses of spatial patterns

First, we estimated covariances between boldness and space use (core area, home range), as well as boldness and microhabitat characteristics (ground cover, maximum vegetation height) using bivariate Bayesian mixed effects models (MCMCglmm, Hadfield, 2010). Second, we modelled covariances between boldness and the number of neighbouring individuals (conspecifics and heterospecifics of similar or dissimilar niche groups) within empirically estimated core areas and home ranges. Each model included boldness and one other trait (space use or interaction) as dependent variables, with individual ID and study site as random intercepts, and species and sex as fixed effects. We set weakly informative priors by dividing phenotypic variance by the number of random effects (*ν* = 1, Hadfield, 2010). Variables measured once per individual (e.g. space use, interaction counts) were modelled with fixed within‐individual variance (0.0001) and zero within‐individual covariance (Dingemanse & Dochtermann, 2013; Houslay & Wilson, 2017). Gaussian distributions were used for continuous traits, and a Poisson distribution for counts. Models ran for 101,000 iterations (burn‐in: 1000; thinning: 100), ensuring low autocorrelation. We extracted species‐specific covariances and 95% credible intervals. Following the language‐of‐evidence framework of (Dingemanse et al., 2020; Muff et al., 2022), we interpreted covariances as strong, moderate, or no association based on whether intervals excluded, skewed from, or centred around zero. Correlation coefficients were calculated from covariances and standard deviations to facilitate interpretation and comparison across traits.

## 3 RESULTS

### 3.1 Individual behavioural differences and their influence on space use

Behavioural variables showed species‐specific patterns of repeatability, with latency measures being repeatable primarily in the generalists, that is bank voles and striped field mice, whereas activity‐related measures (e.g. crossings and jumps) were repeatable (*R* > 0.1) in the specialists, that is common voles and field voles (Table S2). The PCA identified two meaningful components, explaining 78% of the variance in the data (first component: 47%; second component: 31%).

All variables from the open‐field test loaded on the first component (*λ* = 2.83, all loadings > 0.7; Table S3), which we interpreted as a measure of exploration. The second component reflected the latencies from the dark–light test (*λ* = 1.86, all loadings > 0.7; Table S3) and was interpreted as a measure of boldness. Exploration and boldness scores showed little phenotypic correlation (*ρ* = 0.1, *p* = 0.334). Both PCA components were repeatable over time (*R* > 0.16; Table S2). Consequently, low‐high values in boldness score reflect behavioural differences along the shy‐bold continuum. Patterns for exploration differed among species and showed no consistent covariation with spatial metrics at the community level. We therefore focus the main text on boldness, whereas exploration results are summarized in Table 1. This distinction highlights that, even among repeatable behavioural traits, the degree of repeatability does not necessarily reflect their ecological relevance for predicting interactions. Instead, traits that consistently covary with ecological variables across species may be more informative for community‐level inference. Boldness distributions differed among species, with more generalist species exhibiting a broader distribution of scores compared to more specialist species (Figure S1). In particular, very high boldness scores (upper quintile) were more frequent in bank voles and striped field mice than in common and field voles. Community‐level covariance estimates showed that boldness was most strongly associated with space‐use variables, whereas exploration exhibited mostly weak or inconsistent associations across the same set of spatial metrics (Table 1).

**TABLE 1:** Community‐level covariances (posterior modes) between personality axes and spatial metrics derived from telemetry‐based individual‐specific space‐use areas.

Variable	Boldness			Exploration		
	Cov.	CI low	CI up	Cov.	CI low	CI up
Core area size	**0.65**	**0.28**	**0.83**	−0.20	−0.56	0.40
Home range size	**0.63**	**0.24**	**0.85**	−0.01	−0.49	0.46
Ground cover	***0.36***	***−0.13***	***0.73***	0.20	−0.49	0.57
Vegetation height	***−0.55***	***−0.78***	***0.05***	−0.08	−0.53	0.56
Intraspecific overlap (core)	***0.15***	***−0.34***	***0.60***	***−0.11***	***−0.58***	***0.29***
Intraspecific overlap (home)	***−0.09***	***−0.62***	***0.36***	***−0.16***	***−0.55***	***0.37***
Interspecific overlap (core)	***−0.27***	***−0.55***	***0.34***	0.02	−0.49	0.41
Interspecific overlap (home)	**0.60**	**0.00**	**0.76**	***−0.51***	***−0.74***	***0.03***
Track length	**0.65**	**0.01**	**0.81**	***−0.36***	***−0.72***	***0.21***
*Neighbour counts—Core area*						
No. conspecifics	0.05	−0.58	0.47	0.11	−0.50	0.58
No. heterospecifics	−0.08	−0.42	0.57	−0.07	−0.48	0.49
Same‐niche neighbours	0.15	−0.47	0.53	0.04	−0.47	0.53
Same‐niche heterospecifics	***0.23***	***−0.37***	***0.66***	0.01	−0.61	0.49
Different‐niche heterospecifics	0.14	−0.43	0.59	0.18	−0.47	0.54
*Neighbour counts—Home range*						
No. conspecifics	0.16	−0.53	0.50	−0.04	−0.48	0.51
No. heterospecifics	−0.08	−0.46	0.53	0.02	−0.51	0.47
Same‐niche neighbours	0.10	−0.51	0.50	−0.04	−0.49	0.49
Same‐niche heterospecifics	0.04	−0.44	0.57	0.06	−0.50	0.48
Different‐niche heterospecifics	0.16	−0.48	0.56	−0.05	−0.47	0.50
*Spatial interaction metrics*						
Distances to conspecifics	***−0.19***	***−0.40***	***0.16***	***0.22***	***−0.20***	***0.47***
Distances to heterospecifics	***0.20***	***−0.20***	***0.35***	−0.07	−0.41	0.30
Nearest neighbour (general)	0.07	−0.32	0.32	***0.27***	***0.00***	***0.61***
Nearest conspecific neighbour	***−0.23***	***−0.50***	***0.29***	***0.24***	***−0.21***	***0.60***
Nearest heterospecific neighbour	***0.32***	***−0.11***	***0.58***	***−0.14***	***−0.49***	***0.27***

*Note*: Values are shown with 95% credible intervals. Strongly supported effects are shown in bold; weak to moderately supported effects are shown in bold italics; non‐supported effects are shown in regular font (Dingemanse et al., 2020; Muff et al., 2022).

Based on radio‐tracked individuals (n = 54), there was strong evidence for a positive covariation between boldness and core and home range size, respectively (Table 1, Figure 2). Associations with microhabitat characteristics were weaker, but boldness showed moderate support for positive covariation with ground cover and negative covariation with vegetation height (Table 1, Figure S2). In contrast, exploration showed no consistent community‐level associations with these space‐use variables (Table 1).

**FIGURE 2:**
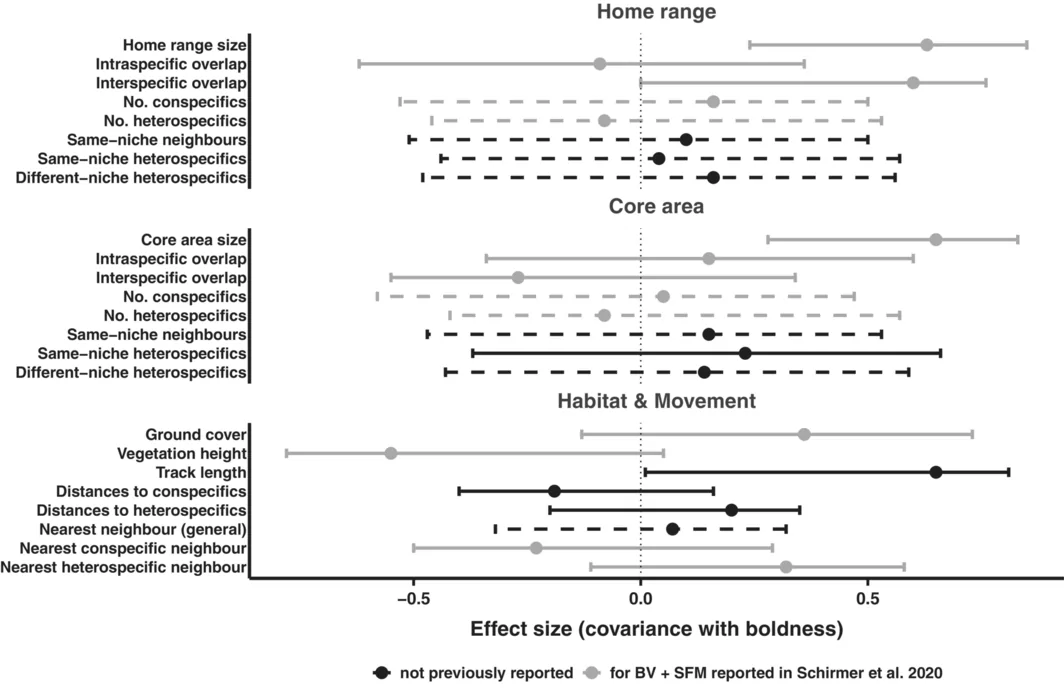
Effect plot summarizing posterior among‐individual correlation estimates between boldness and space use in: (i) individual home ranges, (ii) core areas, and (iii) habitat use and movement metrics including distances to their neighbours (top to bottom). Points show posterior‐mode correlation estimates and horizontal lines indicate associated 95% credible intervals; the vertical dotted line denotes zero correlation. Effects classified as weakly, moderately, or strongly supported following the language‐of‐evidence framework (Dingemanse et al., 2020; Muff et al., 2022) are shown with solid interval lines, whereas effects classified as no effect are shown with dashed interval lines. Results previously reported for bank voles and striped field mice in (Schirmer et al., 2020) are shown in dark grey; results not previously reported are shown in black.

### 3.2 Spatial encounter structure within and between species

Analyses based on empirically estimated space‐use areas provided weak to moderate evidence for covariation between boldness and the number of neighbouring heterospecifics of the same niche within individual core areas, as well as for covariation with distances to conspecifics and heterospecifics (Table 1). At all spatial scales, there was no evidence that sex affected the number of neighbouring individuals or distances to other individuals, regardless of their ecological similarity (Table S7).

At the home range scale, there was no clear evidence for covariation between boldness and neighbour count metrics (Table 1). Although some posterior estimates were slightly shifted away from zero, credible intervals broadly overlapped zero for conspecifics, heterospecifics, same‐niche neighbours, same‐niche heterospecifics, and different‐niche heterospecifics alike. Within home ranges, all neighbour‐count metrics were classified as showing no clear effect of boldness at the community level (Table 1, Figure 2).

Within core areas, most neighbour‐count metrics showed no clear covariation with boldness at the community level (Table 1). The only neighbour metric showing evidence of association was the number of same‐niche heterospecifics within the core area, which tended to increase with boldness (Table 1; Figure 3). The numbers of conspecifics, heterospecifics in general, same‐niche neighbours overall, and different‐niche heterospecifics showed no clear association with boldness.

**FIGURE 3:**
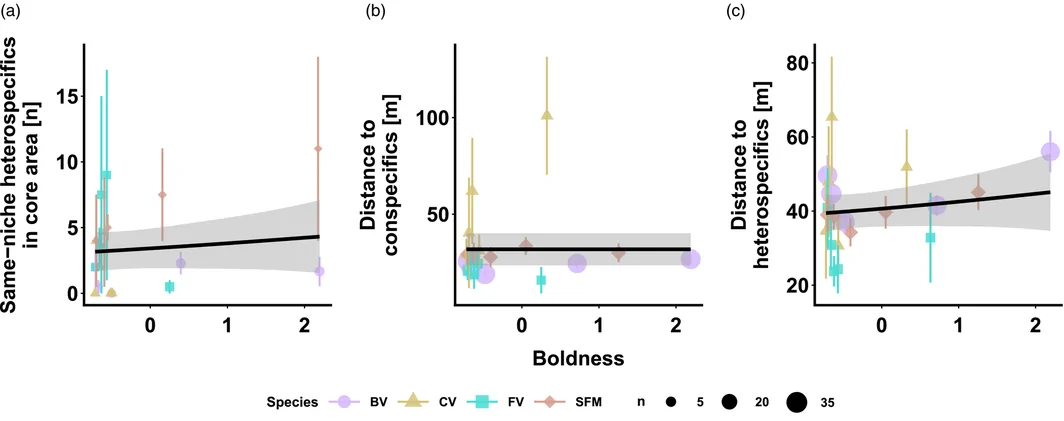
Relationships between individual boldness and spatial interaction metrics: (a) the number of ecologically similar heterospecific individuals within the focual individual's core area, (b) distance to conspecific individuals, and (c) distance to heterospecific individuals. Points show species‐specific quintile means (Q1–Q5) of the response variable plotted against the mean boldness score within each quintile (original boldness scale). Error bars indicate ±SE and point size reflects the number of observations per quintile. Species are colour‐coded (BV, CV, FV, SFM). The black curve shows a pooled smooth fitted to the raw (non‐binned) data across all species within each panel, with a shaded 95% confidence interval.

Distance‐based metrics provided stronger evidence for personality‐dependent spatial structuring than neighbour‐count metrics (Table 1, Figure 2). We found weak evidence for negative covariation between boldness and distances to conspecifics and weak positive covariation with distances to heterospecifics. Similarly, there was weak evidence for negative covariation between boldness and nearest conspecific distance, whereas nearest heterospecific distance showed moderate positive covariation with boldness. By contrast, there was no evidence for an association between boldness and nearest neighbour distance in general.

Overlap metrics showed mixed levels of support (Table 1). We found evidence for a weak negative covariation of Boldness with intraspecific overlap in home ranges, a weak positive covariation with intraspecific overlap in core areas, and a weak negative covariation with interspecific overlap in core areas, but a strong positive covariation with interspecific overlap in home ranges.

## 4 DISCUSSION

By combining previously analysed tracking data with newly analysed species and community‐level models, we assessed space use and spatial interactions among most community members. Across the rodent community, boldness showed its strongest associations with space‐use variables, whereas covariation with neighbour‐count metrics was generally weaker and more context dependent. Most neighbour‐count metrics showed no clear relationship with boldness, although some same‐niche and distance‐based measures indicated weak to moderate evidence of personality‐dependent spatial structuring.

This suggests that the role of intraspecific trait variation in shaping spatial encounter patterns is dependent on ecological similarity between species. Where personality‐dependent encounter patterns emerged, they were more consistent with expectations for ecologically similar competitors than for ecologically dissimilar ones, suggesting that ecological similarity, and thus the potential for competition, modulates how behavioural variation translates into spatial encounter structure. Moreover, these patterns were only detectable when analyses accounted for individual variation rather than relying on species mean estimates alone. This finding underscores that differences in space use and spatial interactions are more pronounced at the individual level and highlights the importance of incorporating individual variation into studies on space use and species interactions. Importantly, our spatial metrics quantify encounter potential rather than realized competition, because resource availability and habitat structure may influence whether spatial proximity translates into competitive interactions. Consistent with this interpretation, personality‐dependent spatial structuring was most pronounced among ecologically similar, more generalist species pairs, whereas it appeared weaker among more specialized, ecologically distinct species combinations.

### 4.1 General space use patterns

At the community level, boldness showed strong positive covariation with both core area and home range size. Further, we found moderate support for boldness positively covarying with ground cover and negatively with vegetation height (Figure S2). Behavioural type–dependent space use is therefore not only evident in the more generalist species pair, as shown before (Schirmer et al., 2019, 2020), but also in the more specialist species pair (Erixon et al., 2025). Together, these results suggest that behavioural type–dependent space use and microhabitat use may represent a general feature of natural rodent communities and may contribute to individual niche differentiation. Behavioural type–dependent space use was also reported in chipmunks (Boyer et al., 2010), burbots (Harrison et al., 2015), hares (Stiegler et al., 2022), lizards (Spiegel et al., 2015), and fish (Kobler et al., 2009), suggesting that individual niche differentiation by behavioural type–dependent space use, for example through differential use of predation‐free space and risk‐related microhabitats, might be a widespread ecological phenomenon also at the community level. Importantly, personality structure differed among species, with some species showing stronger repeatability in exploration than in boldness. However, only boldness showed consistent associations with spatial behaviour across species, indicating that different behavioural axes do not contribute equally to spatial ecological processes.

### 4.2 Interactions between species depend on ecological similarity

Community‐level analyses provided only limited evidence that boldness structured the composition of neighbouring individuals, but where such effects emerged, they were more consistent for ecologically similar competitors than for ecologically dissimilar ones. Weak community‐level effects may partly reflect species‐specific differences in territoriality, habitat use, and movement mode, which could modulate how boldness translates into local encounter structure. Because these rodents predominantly compete via exploitation (Jancewicz & Gliwicz, 2017; Kozakiewicz & Boniecki, 1994; Myllymäki & Myllymaki, 1977), ecological similarity is a plausible determinant of how strongly personality‐dependent spatial positioning influences spatial interaction structure. More generalist species with broader ecological overlap may therefore show stronger scope for behavioural variation to redistribute potential encounters among competitors compared to more specialist species.

By contrast, the more specialist species pair showed little evidence that boldness systematically altered heterospecific encounter structure. One possible explanation is that competition may be weaker or structured differently in these species, either because key resources are less limiting or because their movement ecology reduces direct spatial encounters. Interestingly, the reduced frequency of highly bold individuals in the more specialist species pair is consistent with the hypothesis that strong behavioural differentiation may be less favoured under conditions of lower interspecific competition. However, because telemetry sample sizes for common voles were small, we cannot exclude the possibility that subtle behavioural effects remained undetected. Nonetheless, posterior distributions were centred near zero, suggesting strong evidence for the absence of an effect rather than a power limitation in detecting it. (Dingemanse et al., 2020; Muff et al., 2022). In addition, the more constrained movement ecology of *Microtus* voles may reduce the frequency with which differences in boldness translate into measurable variation in encounter structure.

Our data show that species with broader niches might face a broader range of competitors within a community due to their flexibility in resource use. Higher levels of individual variation might therefore be beneficial for adjusting to the wider range of competitors and, consequently, facilitating coexistence. At the species level, an increased individual niche specialization could increase the total number of competitors a species can coexist with. In many rodent species, natural population densities are highly dynamic and fluctuate strongly within and between years (Hansson, 1979; Korpimäki, 1993; Korpimäki et al., 2002; Krebs et al., 2010). With these density fluctuations, the proportion of each species within a community most likely changes as well. Consistent interindividual differences might only come into play in interactions of individuals from similar ecological niches with high population densities, leading to high levels of interspecific competition. Moreover, our data show that the two highly abundant generalist species expressed a higher level of variation in boldness compared to the less abundant fossorial vole species (Figure S1), indicating that population densities might also be connected to the amount of intraspecific behavioural variation. Increasing population densities might favour within‐species behavioural variation (Milles et al., 2022), thereby serving as a mechanism of reducing both intraspecific and interspecific competition.

By contrast, ecologically dissimilar heterospecifics showed no clear covariation with boldness, consistent with the expectation that lower niche overlap weakens the extent to which personality‐dependent spatial positioning affects interaction opportunities. Individuals may distribute spatial encounters differently along a gradient of competitor similarity, reflecting variation in how behavioural types interact with competitors of differing ecological similarity. With boldness being positively associated with an individual's competitive ability in intraspecific encounters (Erixon et al., 2024), this indicates an unbalanced competitive strength between behavioural types (Cote et al., 2010; Duckworth, 2008; Duckworth & Badyaev, 2007; Webster et al., 2009). Further, our results indicate that behavioural types may differ in how they encounter competitors depending on the ecological similarity of those competitors. In the more specialist species pair, the absence of boldness‐related covariation with heterospecific encounter metrics indicates that boldness may be less important for structuring spatial interactions in these species than in the more generalist pair.

### 4.3 Relative importance of individual variation for ecological interactions

Modern coexistence theory distinguishes stabilizing mechanisms, which promote niche differentiation, from equalizing mechanisms, which reduce average fitness differences among species (Adler et al., 2007; Chesson, 2000b). Our study was not designed to quantify niche partitioning or fitness directly, and therefore cannot test these mechanisms explicitly. However, consistent individual behavioural differences are increasingly recognized as important sources of ecological variation within populations (Bolnick et al., 2003, 2011; Violle et al., 2012). In this study, behavioural variation was associated with aspects of spatial encounter structure, particularly for some measures involving ecologically similar competitors. Such non‐random encounter patterns can modify interaction networks within communities and thereby influence ecological processes that underlie coexistence dynamics (Bolnick et al., 2003; Violle et al., 2012). Rather than demonstrating stabilizing or equalizing mechanisms directly, our results identify behavioural variation as a potential contributor to the spatial structuring of interactions that may ultimately affect those mechanisms. This interpretation is consistent with recent work showing that intraspecific trait variation in personality‐related movement behaviour can promote coexistence by reducing interspecific differences in competitive ability (Milles et al., 2020). Although our analyses are correlational and cannot establish causality directly, previous work in this system showed that individuals non‐randomly select habitats according to their behavioural type, while behavioural traits remain relatively stable across habitat contexts (Erixon et al., 2025). This supports the interpretation that personality–dependent habitat selection contributes to observed space‐use patterns, although reciprocal influences between behaviour and environmental conditions cannot be excluded. More broadly, incorporating individual‐level behavioural variation into community ecology can refine predictions derived from species‐level trait approaches because interaction strengths may differ systematically among individuals within the same species (Bolnick et al., 2011; Violle et al., 2012). Future work linking behavioural traits with direct measures of resource use and fitness consequences will be required to determine explicitly how personality variation influences coexistence processes.

Our results suggest that consistent individual differences in behaviour can contribute to individual niche differentiation and may bias encounter probabilities toward particular competitor classes. Conclusively, consistent individual behavioural differences may contribute to processes analogous to stabilizing or equalizing mechanisms, although our data do not directly quantify niche differentiation or fitness differences. At the species level, differentiation among individual niches could broaden population‐level niche width and potentially increase ecological separation among coexisting species (Bolnick et al., 2011; Violle et al., 2012). At the same time, non‐random encounter structure among behavioural types could alter how competitive asymmetries are distributed among individuals. At the species level, differentiation among individual niches could broaden population‐level niche width and potentially increase ecological separation among coexisting species (Bolnick et al., 2011; Violle et al., 2012).

## 5 CONCLUSION

Consistent behavioural differences within species can shape space use and spatial encounter structure, with potential consequences for how interactions are distributed within communities. Where personality‐dependent encounter patterns emerged, they were most consistent for ecologically similar competitors, suggesting that ecological similarity modulates the extent to which behavioural variation is reflected in spatial encounter structure. In contrast, ecologically dissimilar species showed little evidence of personality‐dependent encounter structuring, suggesting that species‐level niche differences may already reduce the scope for individual behavioural variation to modify encounter patterns. Thus, both species‐level and individual‐level niche differentiation contribute to the complex interaction networks found in natural communities. Future studies should integrate both levels to improve our understanding of species interactions across ecological communities, the drivers of coexistence, and biodiversity maintenance.

## AUTHOR CONTRIBUTIONS

Annika Schirmer, Jana A. Eccard, and Melanie Dammhahn developed and designed the study. Jana. A. Eccard developed the tracking methodology. Annika Schirmer and Melanie Dammhahn. collected the data. Annika Schirmer and Jonas Stiegler analysed the data, and all authors discussed the results. Annika Schirmer and Jonas Stiegler wrote the manuscript. All co‐authors contributed critically to the drafts and gave final approval for publication.

## FUNDING INFORMATION

Funding for A.S. was provided by the German Research Foundation (DFG) within the BioMove research training group (grant no. DFG‐GRK 2118/1).

## CONFLICT OF INTEREST STATEMENT

The authors declare no competing interests.

## ETHICS STATEMENT

Experiments were conducted under the permission of the Landesamt fuer Umwelt, Verbraucherschutz und Gesundheit, Brandenburg, Germany (LUGV 7RO‐4610/34+5#86908/2011; V3‐2347‐44‐2011). All applicable institutional and national guidelines for the care and use of animals were followed.

## Supporting information


**Figure S1.** Variation in boldness shown as density curves separated into data‐derived quintiles for all study species.
**Figure S2.** General space‐use patterns of bank voles (BV, *Myodes glareolus*, *n* = 21), striped field mice (SFM, *Apodemus agrarius*, *n* = 15), common voles (CV, *Microtus arvalis*, *n* = 5) and field voles (FV, *Microtus agrestis*, *n* = 13) based on radio telemetry data.
**Table S1.** Qualitative differences in ecological traits among species pairs.
**Table S2.** Repeatability estimates (R) for each behavioural variable across four rodent species based on the individual difference test.
**Table S3.** Results from the principal component analysis (PCA).
**Table S4.** Species‐specific mean core area and home range sizes with corresponding standard deviations (SD). All areas are shown in square meters.
**Table S5.** Effect of boldness on core area and home range size.
**Table S6.** Covariances for general space‐use patterns with boldness.
**Table S7.** Effects of the factor sex on general space use patterns of the four study species calculated via Bayesian mixed effect models.

## Data Availability

Data available from the Dryad Digital Repository https://doi.org/10.5061/dryad.2rbnzs85c (Stiegler et al., 2026).
