# Supplementary material for: Boldness and niche width structure spatial interactions in a natural rodent community
Source: J Anim Ecol. 2026 Jul 17;95(10):1999–2012. doi: 10.1111/1365-2656.70320 (PMC13643188; doi:10.1111/1365-2656.70320)
Supplement: Supplementary file 1 [file JANE-95-1999-s001.pdf]

Boldness and Niche Width Structure Spatial Interactions in a Natural Rodent Community

Contents

1 Supporting Information **2**

1.1 Variation in boldness across study species . . . 2

1.2 Boldness-dependent space use patterns . . . 3

1.3 Supporting tables . . . 4

1.3.1 Differences in ecological traits among species pairs . . . 4

1.3.2 Repeatabilities of variables from the individual difference test for each species . . . 5

1.3.3 Principal component analysis (PCA) . . . 6

1.3.4 Species-specific mean core area and home range sizes . . . 6

1.3.5 Effect of boldness on core area and home range size . . . 6

1.3.6 Covariances for general space use patterns with boldness . . . 7

1.3.7 Effects of sex on general space use patterns . . . 7

1 Supporting Information

1.1 Variation in boldness across study species

For visualization purposes, individuals were grouped into data-derived quintiles based on their boldness score. The variation of boldness scores across individuals of all study species and the resulting quintile bins are shown in Fig. S1.

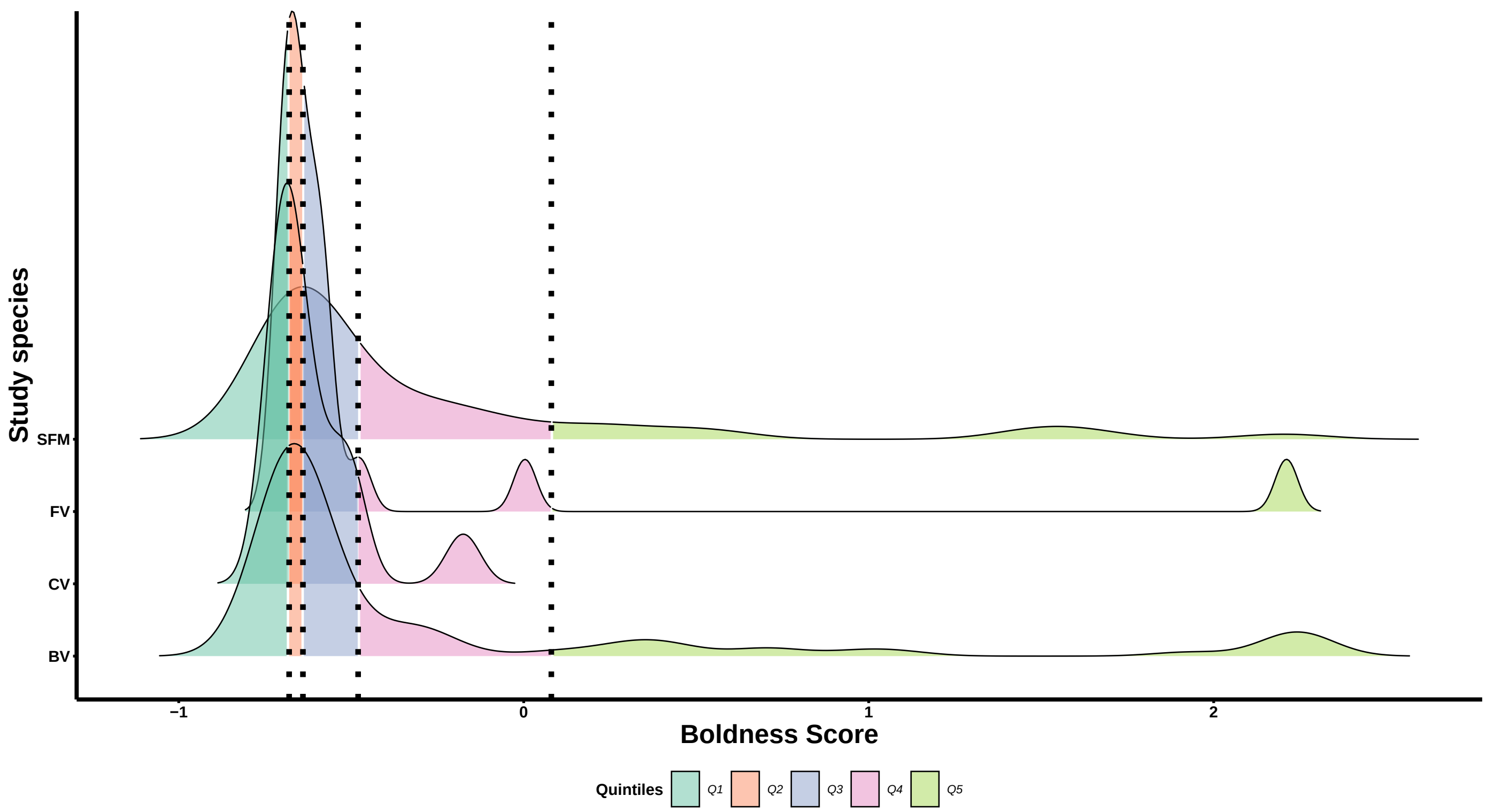

Figure S1: Variation in boldness shown as density curves separated into data-derived quintiles for all study species. Represented are mean boldness scores for each individual, based on the scores from the PCA component. Shown are bank voles (BV, *Myodes glareolus*, $n = 82$), striped field mice (SFM, *Apodemus agrarius*, $n = 53$), common voles (CV, *Microtus arvalis*, $n = 14$) and field voles (FV, *Microtus agrestis*, $n = 19$). The height of the curve represents the relative sample size, and the vertical dashed lines separate the color-coded quintiles.

1.2 Boldness-dependent space use patterns

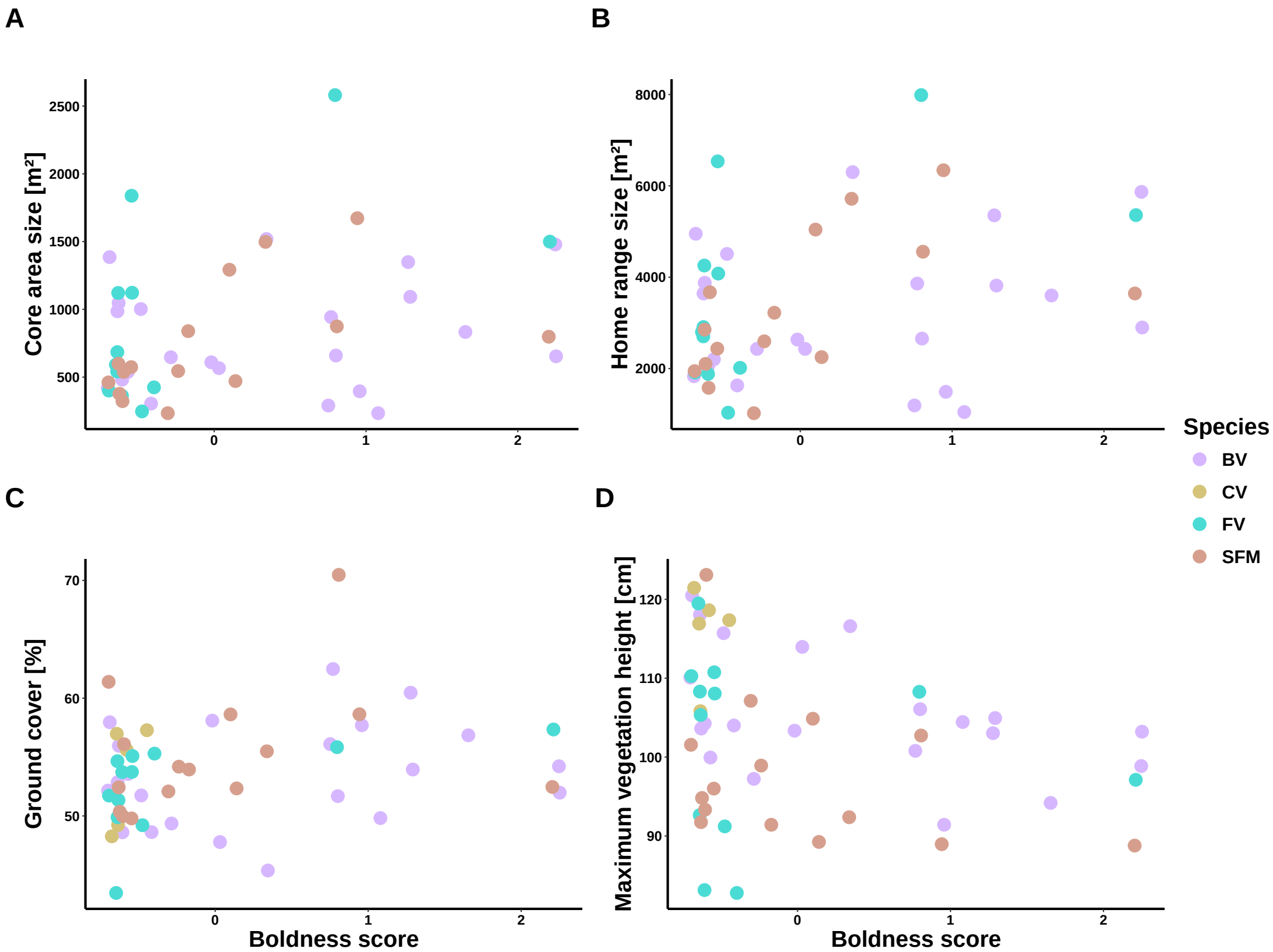

Figure S2: General space use patterns of bank voles (BV, *Myodes glareolus*, $n = 21$), striped field mice (SFM, *Apodemus agrarius*, $n = 15$), common voles (CV, *Microtus arvalis*, $n = 5$), and field voles (FV, *Microtus agrestis*, $n = 13$) based on radio telemetry data. Data points for significant covariances are shown for A: Core area size (BV, FV, SFM), B: Home range size (BV, FV, SFM), C: Ground cover (BV, CV, FV, SFM), and D: Maximum vegetation height (BV, CV, FV, SFM).

1.3 Supporting tables

1.3.1 Differences in ecological traits among species pairs

Table S1: Qualitative differences in ecological traits among species pairs. A value of 1 indicates that species differ in the respective trait; empty cells indicate broadly similar trait expression. The column sum reflects the number of trait differences between species pairs. Traits are based on published descriptions of habitat use, diet, and spatial behavior (see references below).

Species pair	Species	Diet breadth	Habitat use	Home range size	Vegetation structure	Sum
	Mg-Aa	0	0	0	0	0
	Mg-Marv	1	1	1	1	4
	Mg-Magr	1	1	1	1	4
	Aa-Marv	1	1	1	1	4
	Aa-Magr	1	1	1	1	4
	Marv-Magr	0	0	0	0	0

Trait definitions: Diet breadth refers to the degree of omnivory versus specialization on grass-dominated food resources. Habitat use describes differences in vertical or structural habitat use (e.g., above-ground versus dense grassland/runway systems). Home range size refers to relative differences in space use reported in the literature. Vegetation structure refers to differences in preferred ground cover and habitat complexity. (Holišová, 1971; Kozakiewicz and Boniecki, 1994; Eccard et al., 2002; Myllymäki and Myllymaki, 1977; Szalay et al., 2000; Schirmer et al., 2019)

Sources

Eccard, J. A., Ylönen, H., Eccard, J. A., and Ylönen, H. (2002). Direct interference or indirect exploitation? an experimental study of fitness costs of interspecific competition in voles. *Oikos*, 99:580–590.

Holišová, V. (1971). The food of clethrionomys glareolus at different population densities. *Přírodovědné Práce Československé Akademie Věd (Nová Série)*, 5:1–43.

Kozakiewicz, A. and Boniecki, P. A. (1994). Intra-and interspecific behaviours in bank vole and striped-field mouse under enclosure conditions. *Acta Theriologica*, 39:29–36.

Myllymäki, A. and Myllymaki, A. (1977). Interactions between the field vole microtus agrestis and its microtine competitors in central-scandinavian populations. *Oikos*, 29:570.

Schirmer, A., Herde, A., Eccard, J. A., and Dammhahn, M. (2019). Individuals in space: personality-dependent space use, movement and microhabitat use facilitate individual spatial niche specialization. *Oecologia*, 189:647–660.

Szalay, F. S., Mitchell-Jones, A. J., Amori, G., Bogdanowicz, W., Kryštufek, B., Reijnders, P. J. H., Spitzenberger, F., Stubbe, M., Thissen, J. B. M., Vohralik, V., and Zima, J. (2000). The atlas of european mammals. *The Quarterly Review of Biology*, 75:472.

1.3.2 Repeatabilities of variables from the individual difference test for each species

Table S2: Repeatability estimates (R) for each behavioral variable across four rodent species based on the individual difference test. The table shows the repeatability value (R), the lower (CI low) and upper (CI up) bounds of the $95\,\%$ confidence interval, and the p-value based on permutation tests. Significant repeatabilities ($p < 0.05$) are marked in bold.

Species	Variable	R	CI low	CI up	p
Bank vole	Lat_head	**0.28**	0.096	0.455	**0.003**
	Lat_body	**0.32**	0.135	0.497	**0.002**
	Lat_center	0.06	0.000	0.232	0.242
	Jumps	0.07	0.000	0.260	0.228
	Crossings	0.03	0.000	0.213	0.401
	Sections	0.11	0.000	0.292	0.114
	Activity	0.00	0.000	0.165	1
Striped field mouse	Lat_head	0.17	0.000	0.400	0.074
	Lat_body	**0.27**	0.043	0.485	**0.014**
	Lat_center	0.08	0.000	0.304	0.226
	Jumps	**0.20**	0.000	0.403	**0.046**
	Crossings	**0.21**	0.000	0.441	**0.030**
	Sections	0.03	0.000	0.263	0.364
	Activity	0.01	0.000	0.229	0.492
Common vole	Lat_head	0.23	0.000	0.628	0.129
	Lat_body	0.18	0.000	0.594	0.195
	Lat_center	0.14	0.000	0.581	0.241
	Jumps	**0.82**	0.561	0.927	**0.006**
	Crossings	**0.50**	0.015	0.774	**0.018**
	Sections	0.29	0.000	0.663	0.113
	Activity	0.21	0.000	0.590	0.168
Field vole	Lat_head	0.00	0.000	0.468	1
	Lat_body	0.00	0.000	0.489	1
	Lat_center	0.00	0.000	0.501	1
	Jumps	**0.50**	0.000	0.779	**0.030**
	Crossings	**0.57**	0.025	0.830	**0.018**
	Sections	0.27	0.000	0.707	0.199
	Activity	0.29	0.000	0.691	0.137

1.3.3 Principal component analysis (PCA)

Table S3: Results from the principal component analysis (PCA). Represented are the highest loadings on the two components for each variable, as well as the eigenvalues of the components and the proportional and cumulative amount of variance explained by the components.

Variable	Component 1	Component 2
Lat_head		0.964
Lat_body		0.961
Lat_center	0.797	
Crossings	0.847	
Sections	0.900	
Activity	0.815	
Eigenvalues	2.826	1.857
Proportion variance	0.471	0.309
Cumulative variance	0.471	0.781

1.3.4 Species-specific mean core area and home range sizes

Table S4: Species-specific mean core area and home range sizes with corresponding standard deviations (SD). All areas are shown in square meters.

Species	Mean core area	SD core area	Mean home range	SD home range
Myodes glareolus	792.34	397.89	3194.72	1510.18
Microtus arvalis	1050.41	151.58	4566.14	850.65
Microtus agrestis	945.56	683.15	3641.11	2010.32
Apodemus agrarius	739.59	432.58	3264.73	1556.99

1.3.5 Effect of boldness on core area and home range size

Table S5: Effect of boldness on core area and home range size. Shown are the posterior mean (post.mean), the lower and upper limit of the $95\,\%$ confidence interval (CI low, CI up), the effective sample size (eff.samp), and the MCMC p-value (pMCMC).

Variable	Factor	post.mean	CI low	CI up	eff.samp	pMCMC
Core area size	Boldness	204.70	47.05	351.16	1000	$<$0.001
Home range size	Boldness	698.50	222.70	1246.50	1000	$<$0.001

1.3.6 Covariances for general space use patterns with boldness

Table S6: Covariances for general space use patterns with boldness. Represented are the covariance value and the belonging lower (CI low) and upper (CI up) limits of the $95\,\%$ credibility interval. Covariances with credibility intervals significantly different from zero are marked in bold.

	Covariance	CI low	CI up
**Bank vole**			
Core area size	**0.28**	**0.031**	**0.746**
Home range size	**0.35**	**0.275**	**0.801**
Ground cover	**0.28**	**0.044**	**0.776**
Vegetation height	**-0.26**	**-0.053**	**-0.675**
**Striped field mouse**			
Core area size	**0.17**	**0.055**	**0.762**
Home range size	**0.23**	**0.056**	**0.795**
Ground cover	**0.20**	**0.059**	**0.781**
Vegetation height	**-0.40**	**-0.045**	**-0.840**
**Common vole**			
Core area size	0.18	-0.010	0.443
Home range size	0.16	-0.010	0.641
Ground cover	**0.18**	**0.006**	**0.961**
Vegetation height	**-0.34**	**-0.075**	**-0.932**
**Field vole**			
Core area size	**0.76**	**0.003**	**0.910**
Home range size	**0.67**	**0.002**	**0.911**
Ground cover	**0.61**	**0.038**	**0.906**
Vegetation height	**-0.32**	**-0.163**	**-0.807**

1.3.7 Effects of sex on general space use patterns

Table S7: Effects of the factor sex on general space use patterns of the four study species calculated via Bayesian mixed effect models. Represented are the posterior mean, lower (CI low) and upper (CI up) limit of the $95\,\%$ confidence interval, effective sample size, and p-value.

Variable	Factor	post.mean	CI low	CI up	eff.samp	pMCMC
Core area size	sex	-4.28	-58.146	52.118	1000	0.874
Home range size	sex	-15.06	-198.535	183.048	1099	0.856
Ground cover in home range	sex	-0.18	-0.953	0.549	1000	0.614
Max. Vegetation height in home range	sex	-0.09	-1.419	1.258	957	0.902
